# Natural and Conventional Cosmetics—Mercury Exposure Assessment

**DOI:** 10.3390/molecules26134088

**Published:** 2021-07-05

**Authors:** Aleksandra Podgórska, Anna Puścion-Jakubik, Anita Grodzka, Sylwia K. Naliwajko, Renata Markiewicz-Żukowska, Katarzyna Socha

**Affiliations:** Department of Bromatology, Faculty of Pharmacy with the Division of Laboratory Medicine, Medical University of Białystok, Mickiewicza 2D Street, 15-222 Białystok, Poland; apodgorska1@student.umb.edu.pl (A.P.); aegrodzka@gmail.com (A.G.); sylwia.naliwajko@umb.edu.pl (S.K.N.); renmar@poczta.onet.pl (R.M.-Ż.); katarzyna.socha@umb.edu.pl (K.S.)

**Keywords:** mercury, natural cosmetics, conventional cosmetics, face, body

## Abstract

Mercury (Hg) can enter the human body through the respiratory tract and digestive system, but also through the skin. Sources of Hg in the environment can be natural processes, but also human activities, including agriculture, chemical, and pharmaceutical industries. Hg can also enter the body through food, but also with cosmetics that are used for a long time. Therefore, the aim of this study was to evaluate the Hg content in 268 randomly selected cosmetics: Natural and conventional, for face and body. Hg content was determined using an atomic absorption spectrometer (AMA 254, Leco, Prague, Czech Republic). It was shown that the face preparations were characterized by a significantly higher Hg content than the body preparations. No differences in the content of the tested element were found between natural and conventional preparations. Hg could be detected in all samples with concentrations measured from 0.348 to 37.768 µg/kg.

## 1. Introduction

Mercury (Hg) has been known and used by man for centuries, such as by Egyptians, Greeks, Romans, and Hindus since ancient times. Currently, despite the existence of restrictions on the use of Hg, it is used in medicine, agriculture, and industry, including the cosmetics industry [1,2].

In nature, Hg occurs as metallic Hg and in the form of organic (e.g., methylmercury and ethylmercury) and inorganic compounds (e.g., Hg chlorides, sulfates, or nitrates) [1,2].

Hg enters the human body primarily through the respiratory system, alimentary tract, and also through the skin. Its absorption depends primarily on the form in which it occurs. Metallic Hg vapors are absorbed by the respiratory system in about 80%. Organic and inorganic compounds are absorbed through the digestive tract, through the skin, and through the sweat and sebaceous glands of the skin [3].

The main source of Hg in the environment is from natural processes, e.g., volcanic eruptions or weathering of rocks, and human activity, which includes, among others, chemical and pharmaceutical industries, agriculture, and coal-fired heating and power plants [2,3,4]. Data on the monitoring of Hg content in soils and sediments have been published for many countries, as well as collected in collective summaries [5]. The increased content is due to local industrial pollution. For example, soils near the city of Qingzhen, China, had a maximum Hg content of 328.95 ppm. This was due to irrigation with river water contaminated with sewage [6]. Data from Poland indicates a content of 62–393 mg/kg [7].

Important sources of exposure are also: a diet rich in marine and freshwater fish [8], amalgam fillings used in dentistry - especially dangerous in some subpopulations [9], and cosmetic products [10].

The permissible Hg contamination depends on the national regulations. Hg in cosmetic products can be an impurity but can also be intentionally added as preservatives: organic Hg compounds such as Thiomersal and phenylmercury salts (along with borate). The Hg contamination of cosmetics occurs during their production or as the result of improper cleaning of the raw materials used. The presence of Hg in cosmetic products, with two exceptions, Volpar and Thiomersal, is not permitted by law. Its trace amount in products can occur as contamination. Thiomersal and phenylmercuric salts may only be added to eye products at a maximum concentration of 0.007%, expressed as Hg. Cosmetics containing both of these compounds must be labeled with ‘contains thiomersal’ or ‘contains phenylmercury compounds’ [11,12,13]. According to US legislation, Hg must not be present in preparations, except in eye products—this element must not exceed 65 parts per million (ppm) in the finished product. It can only be used when no other effective and safe preservative is available. Moreover, Hg is not allowed in any other cosmetic product except in trace amounts below 1 ppm, and only when its presence is unavoidable in accordance with good manufacturing practice (GMP) [14].

For example, in one publication regarding creams from Jamaica, the maximum content was 17,547 mg/kg [15], while in China, Japan, Sri Lanka, Taiwan, Thailand, and the United States, 6% of samples contained Hg above 1000 mg/kg [16]. The highest value characterized cream lightening and lifting purchased in-store in Thailand (45,622 ± 322 mg/kg) [16]. Literature data indicate a high Hg content in cosmetics used to lighten the skin—Hg salts inhibit the formation of melanin, resulting in a lighter skin tone—sample result obtained in publications was 25.7 mg/kg [17]. A study published by Chen et al. (2020) showed that HgCl_2_ has the ability to directly inhibit tyrosinase, which explains the mechanism of action and toxicity of Hg [18].

In addition, the United States Environmental Protection Agency (US EPA) has published a guide to avoiding exposure to Hg from various sources, including creams, which may contain, for example, the term “anti-aging” or “skin lightening” [19].

RAPEX data is also very important. They are the result of non-food surveillance in the EU, where products with very high Hg content have been identified. Laboratories in national authorities carry out routine surveillance of over several hundred products that are on the EU market. Only the most dangerous products detected by random samples are reported under RAPEX, these results reflect exposure, but the scale of the problem appears to be much larger. For example, on 16 March 2021, a report was published on the lightening cream for the face, which contained Hg in an amount of 15,590 mg/kg [20]. The results published by Klaschka (2017) are also very disturbing. The author concluded that over 10 years, data on 724 cosmetic products were placed in the RAPEX system. The maximum recorded Hg content was as much as 42 g/kg (42,000 mg/kg) [21]. This result shows that controls by authorities in EU countries are very important.

The symptoms of Hg poisoning may appear in several organ systems and organs, including the skin. They include redness, rash, facial swelling, and excessive sweating. The changes also affect the hair, which becomes dry, brittle, thin, and dull, and its growth stops [22,23].

According to the European packaging directive, the sum of lead, cadmium, Hg, and chromium (VI) together may not exceed 100 mg/kg in packaging material [24].

The Hg content can be determined using various methods—they differ in the detection limit: atomic absorption apectrometry (limit of detection: 0.1 ng), atomic emission spectrometry (0.005 ng), mass spectrometry (0.005 ng), colorimetry (100 ng), neutron activation analysis (0.01 ng), X-ray fluorescence spectrometry (25 ng), and electron-capture detection spectrometry (0.5 ng) [25,26,27,28,29,30].

Despite the fact that companies conduct many campaigns regarding the safe use of cosmetics and comply with the requirements of GMP, Hg and its compounds still appear in cosmetic products, including those of the highest quality [31]. Therefore, the aim of this study was to assess the Hg content in random cosmetics both from natural and conventional origins. To the best of our knowledge, this is the first such comprehensive safety assessment of cosmetics used for face and body care.

## 2. Results

### Hg Content in the Tested Cosmetics

Table 1 shows data on the median content of Hg in the tested cosmetics, taking into account the area of application and form. It was shown that the highest median Hg content was found in eye creams (3.850), quartile 1 (Q1): 0.383, quartile 3 (Q3): 14.271 μg/kg, serums (2.168, Q1: 0.359, Q3: 5.170 μg/kg), and gels (2.005, Q1: 0.170, Q3: 7.964 μg/kg).

**Figure 1:**
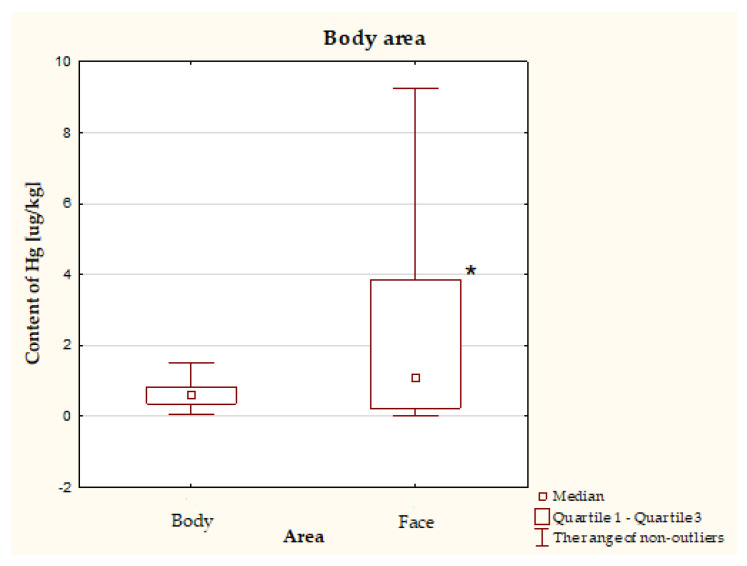
Hg concentration in body (*n* = 134) and face (*n* = 134) cosmetics. * *p* < 0.05.

It was shown that the face care products (median: 1.079 μg/kg, Q1: 0.229 μg/kg, Q3: 3.850 μg/kg) were characterized by a significantly higher Hg content compared to the body care products (median: 0.591 μg/kg, Q1: 0.355 μg/kg, Q3: 0.479 μg/kg) (Figure 1).

**Figure 2:**
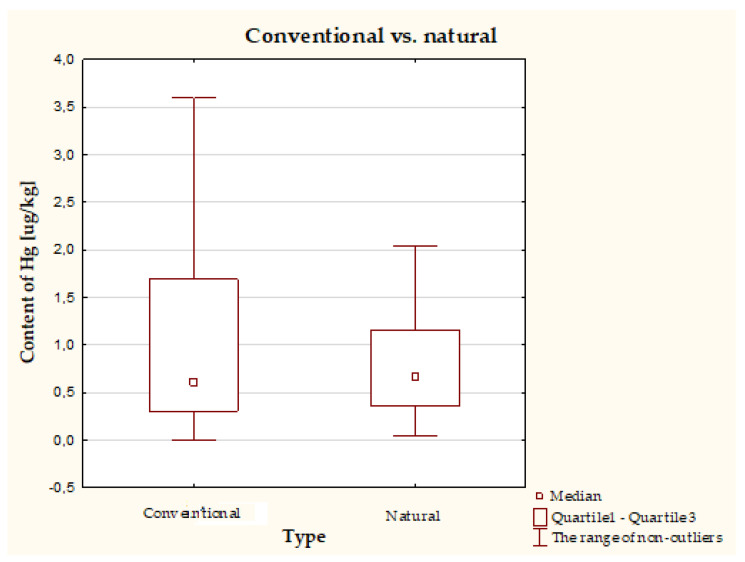
Concentration of Hg in conventional (*n* = 181) and natural (*n* = 87) cosmetics.

Hg content in natural cosmetics (0.672 μg/kg, Q1: 0.357 μg/kg, Q3: 1.150 μg/kg) and conventional cosmetics (0.608 μg/kg, Q1: 0.306 μg/kg, Q3: 1.688 μg/kg) did not differ statistically (Figure 2).

Institutions such as the Food and Drug Administration in the United States of America [14] allow the maximum Hg content in cosmetics to be 1 ppm (i.e., 1000 μg/kg) and only if its presence is unavoidable under GMP. Moreover, Hg compounds are only approved as preservatives in eye care products when there is no better and safer preservative. The concentration must not exceed 65 mg/kg in the finished product. This indicates that the toxic element Hg was detected in all tested cosmetic preparations, i.e., the cosmetics available for sale were in accordance with European or American law.

**Figure 3:**
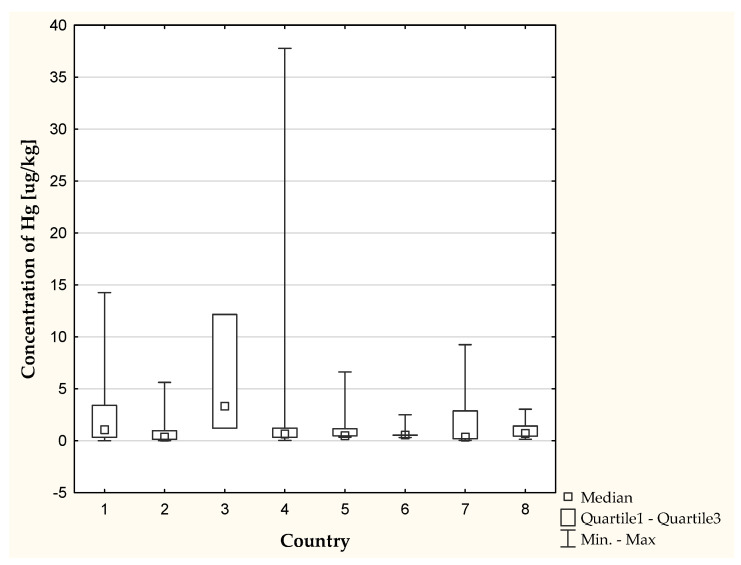
Concentration of Hg depending on the country of origin of the cosmetic (*p* < 0.05). 1: France; 2: Germany; 3: Korea; 4: Poland; 5: Russia; 6: UK; 7: USA; 8: Other.

In addition, we found that the country of origin of a cosmetic has a significant impact on Hg content (Figure 3).

As part of this study, health risk models, including carcinogenic and non-carcinogenic risks, were calculated based on data from the US EPA. The threshold values indicated by this institution were referred to [19]. Chronic exposure to Hg was assessed by calculating chronic daily intake (CDI) and hazard quotient (HQ)—indicating a non-carcinogenic risk [32].

It has been calculated that the CDI for body cosmetics is 8.72 × 10^−12^, and for face cosmetics: 9.56 × 10^−15^. The HQ was: 6.69 × 10^−9^ and 7.36 × 10^−12^, for body and face preparations, respectively.

## 3. Discussion

There are many sources of Hg exposure. The literature emphasizes various ways of human exposure to Hg, e.g., the routes of entry may include the gastrointestinal tract (consumption of large amounts of freshwater and marine food and contaminated dietary supplements), the respiratory tract (as a result of human industrial activities and natural processes), but also the skin (e.g., from the use of Hg-contaminated preparations) [2]. Maximum threshold values written down in various legal instruments are only effective as long as competent authorities control their observation by appropriate surveillance programs and take subsequent action. The rapid alert system RAPEX [20] lists the most serious violations of provisions in non-food consumer products in the European Union, among them also many Hg containing personal care products [21].

Literature reports on the Hg content in cosmetics mainly include studies on the content of this toxic element in skin lightening creams. Data published in RAPEX show an alarming Hg content in skin lightening preparations: incl. 15,590 mg/kg [20], 8061 mg/kg [33] and 7502 mg/kg [34]. This indicates the need to assess the quality of other cosmetics and assess the safety of their use.

Our research found that the Hg content depends on the country of origin of the cosmetic. Studies conducted on face creams (*n* = 6), commercially available in Bangladesh [35], showed that the highest Hg content was 481 ± 9 µg/kg, which was a value almost 13 times higher than the highest results obtained in our research (in serum, 37.768 µg/kg). Studies on cosmetics from France, Italy, Switzerland, and the USA (*n* = 11) showed the content of Hg below limits of quantification (0.16 ng/g) [35]. The study by Peregrino et al. (2011) [36] showed the highest mean Hg content in skin-lightening creams at the level of 875 ± 115 mg/kg. The highest Hg content among skin whiteners cream in Cambodia was 12,590 µg/g (country of origin: China) [37].

In our study, we examined both conventional and natural cosmetics. In recent years, there has been an intense increase in consumer interest in natural cosmetics. The reason is the willingness to take care of health, belief in good recipes, or care for the environment and sustainable development.

The legislation lacks an unambiguous definition of a natural cosmetic, which is why such preparations should meet the standards set by certification institutions. These institutions, such as NATRUE or COSMOS (Cosmetics Organic and Natural Standard), take into account the following issues: use of appropriate quality raw materials and production methods, minimalistic use of substances, and packaging that can be recycled [38].

It should be emphasized that there are no publications comparing the quality of natural and conventional cosmetics, which is why we took up this issue. Interestingly, we showed that although Q3 was higher for conventional cosmetics, the median was higher for natural cosmetics, but this was not statistically significant.

The need to assess the safety of using natural cosmetics is indicated by their prevalence. Kaźmierczak and Wcisło-Dziadecka (2018) [39] conducted a questionnaire on women’s opinions on the use of cosmetics of natural origin. Women from Poland (*n* = 114), aged 17 to 68, were included in the study. The respondents indicated that they use this type of preparation for face care (96.5%), beautifying (94.8%), body (87.9%), and hair care (83.6%). Only half of the women (49.1%) correctly indicated substances that should not be present in cosmetics labeled as natural. Interestingly, the key advantage of using natural cosmetics was the complete reduction or a reduction in the occurrence of allergic reactions [39]. However, literature data indicate that many natural products, including fragrances, are allergens [40]. Our research has shown that all tested preparations, both conventional and natural, meet the requirements for limit values for impurities; the natural preparations were characterized by a similar median Hg content. It should also be emphasized that the face care products were characterized by a significantly higher Hg content, and this type of skin care product is more often chosen by respondents.

The study conducted by Fisher et al. (2017) [41] were aimed at assessing the Hg content in plant raw materials, which are used in cosmetology. The following subjects were evaluated: cottonwood (*Solidago virgaurea* L.), horsetail (*Equisetum arvense* L.), nettle (*Urtica dioica* L.), St. John’s wort (*Hypericum perforatum* L.), wormwood (*Artemisia absinthium* L.), and yarrow (*Achillea millefolium* L.). The authors showed that this content was in the range of 5–28 µg/kg. These values are similar to those shown in cosmetics.

Contamination with the element usually occurs as a result of improper purification of natural raw materials, which are components of cosmetics, and during the production process of cosmetic products. Despite the observance of the principles of GMP, under which numerous production controls are carried out, as well as campaigns for the safe use of cosmetic products, Hg still appears in even high-quality cosmetics. While a single skin contact with a cosmetic containing Hg compounds is usually not associated with more serious side effects, long-term use of these products may lead to their accumulation, causing various health problems [31,35].

The relationship between the presence of Thiomersal and a pseudoallergic skin reaction was investigated by Peng et al. (2019) [42]. The study showed that MrgprB2/MRGPRX2 had an effect on Thimerosal-induced mast cell degranulation as well as the pseudoallergic response in mice—thus, it may play an important role in contact dermatitis in humans.

It should be emphasized that there is an aggregate exposure to Hg through various other products, not only cosmetic preparations. Other sources of exposure include food, packaging materials, clothing, electronics, etc.

Chronic exposure to Hg resulting from the use of cosmetics was assessed by Alam et al. (2019) [32]. The above study was carried out on only six creams frequently used by Bangladeshi people. The authors obtained a result of HQ higher than in our publication (3.49 × 10^−9^). The highest value of the HQ, calculated for a single sample, was 8.67 × 10^−7^, which was a value several times higher than that obtained in this study. An HQ value of less than 1 indicates that there is little risk of exposure to Hg, both in the Bangladeshi population and in the Polish population. Some of the cosmetics we tested came from well-known, large cosmetic companies; hence, the estimate concerns a much larger population.

## 4. Materials and Methods

### 4.1. Materials

The research material consisted of 268 cosmetics, available in stationary and online sales and in pharmacies and cosmetics shops, from Polish and foreign companies. The Hg content of 134 body care preparations and 134 face preparations was assessed.

The products came from different countries: Canada (*n* = 1), China (*n* = 1), Czech Republic (*n* = 2), France (*n* = 47), Germany (*n* = 15), Greece (*n* = 1), Korea (*n* = 3), Norway (*n* = 1), Poland (*n* = 160), Russia (*n* = 5), Spain (*n* = 2), Sweden (*n* = 2), Switzerland (*n* = 1), United Kingdom (*n* = 5), and the USA (*n* = 22).

Among the body preparations the following were tested: body butters (n = 16), body lotions (*n* = 34), body milks (*n* = 22), oils (*n* = 25), peels (*n* = 22), and serums (*n* = 15).

Face care products included: creams (*n* = 46), eye creams (*n* = 11), foams (*n* = 4), gels (*n* = 10), hydrolates (*n* = 3), masks (*n* = 17), micellar fluids (*n* = 9), serums (*n* = 16), creams with SPF (*n* = 13), and tonics (*n* = 5).

Among the cosmetics included in the study, 181 were so-called ‘conventional’ preparations, and 87, ‘natural’.

We selected as many types of cosmetics for the study to best assess the risk of exposure to Hg. All samples did not contain Hg components, as declared by the manufacturers.

### 4.2. Methods

Hg content in face and body care cosmetics was determined using an atomic absorption spectrometer (AMA 254, Leco, Prague, Czech Republic). With this spectrometer, it was possible to determine the total Hg content in liquid and solid samples, regardless of the form of occurrence. It is based on the amalgamation technique. The analyzer relies on the release of Hg from its organic and inorganic compounds, going into its atomic form. The measurement is performed directly; this method does not require prior mineralization of the sample, which is due to the easy formation of the Hg atomic cloud—the pyrolytic mineralization process takes place inside the apparatus. The measurement of the Hg content consists of three steps as described below [43]. Each sample was measured in triplicate, and the results are presented as the mean.

The first step involved drying and then burning the sample (weighing 0.02 g) in a stream of oxygen (duration: 75 s) [43].

The second stage involved passing the released Hg vapors through a catalytic column and then catching them by the amalgamator (150 s) [43].

The third step was to release Hg from the amalgamator and measure its content in measuring cuvettes at a wavelength of 254 nm (45 s) [43].

In order to determine the Hg concentration in the tested cosmetics, a standard curve was made on the basis of a standard Hg solution for atomic absorption spectrometry at a concentration of 1 g/L (Merck, Darmstadt Germany). The curve has five points from 0 to 100 µg/kg.

A method accuracy check was performed each day before starting the analysis and every 10 samples. The method of adding an internal standard of known concentration was used.

The limit of quantification is 0.003 ng Hg. The relative standard deviation was <1.5%.

### 4.3. Evaluation of Chronic Exposure to Hg

The CDIdermal (chronic daily intake dermal) was calculated using the following equation [32]:

$$\mathrm{CDIdermal}=\frac{\mathrm{CS}\times \mathrm{SA}\times \mathrm{AF}\times \mathrm{ABS}\times \mathrm{EF}\times \mathrm{ED}\times \mathrm{CF}}{\mathrm{BW}\times \mathrm{AT}}$$

CS: exposure-point concentration (mg/kg) (for body: 0.677, for face: 3.026);
SA: exposed skin area (cm^2^) (for body: 30,000 cm^2^, for face: 3600 cm^2^);
AF: adherence factor (mg/cm^2^) (0.07);
ABS: dermal absorption fraction (no units) (0.001);
EF: exposure frequency (days/year) (350);
ED: exposure duration (year) (30);
CF: units conversion factor (kg/mg) (10^−6^);
BW: body weight (kg) (70);
AT: mean time for non-carcinogens (days) (25,550).

The HQ (hazard quotient) was calculated using the following formula [30]:

$$\mathrm{HQ}\;=\frac{\mathrm{CDIdermal}}{\mathrm{RfDdermal}}$$

Reference dose (RfDermal) for Hg: 0.0013 mg/kg/day [30].

### 4.4. Statistical Analysis

Statistical analysis was performed using the Statistica 13.3 program (Statsoft, Tibco, Palo-Alto, CA, USA).

The normality of the data distribution was examined by means of the Shapiro–Wilk’s test, Kolmogorov–Smirnov test, and Lilliefors test. The Mann–Whitney U-test was used to show the differences in Hg content between the two groups, and the Kruskal–Wallis ANOVA test between several groups. The differences were assumed to be statistically significant at *p* < 0.05.

## 5. Conclusions

All cosmetic products tested, both conventional and natural, were found to contain Hg above the detection limit. Due to the frequency of application, applying multiple layers, as well as the large body surface area they are applied to, the Hg content of these products should be monitored. Our data lead us to the important final conclusion: on the one hand, it is believed that values below the acceptable standards mean that cosmetics are safe. On the other hand, Hg is a toxic element, so exposure to even a small amount is a hazard to human health. There is no theoretically safe level for this highly toxic element—any concentration above zero is unsafe.

## Figures and Tables

**Table 1:** Hg concentration in the tested cosmetics.

Hg Concentration (μg/kg)					
Type of Cosmetics	*n*	Mean ± SD	Min–Max	Med.	Q1–Q3
**Body Cosmetics**					
Body butter	16	0.848 ± 0.644	0.050–2.127	0.707	0.446–1.164
Body lotion	34	0.683 ± 0.419	0.129–1.716	0.612	0.314–0.854
Body milk	22	0.521 ± 0.404	0.038–1.769	0.428	0.245–0.834
Oil	25	0.903 ± 0.487	0.204–1.974	0.777	0.536–1.208
Peeling	22	0.537 ± 0.204	0.218–0.942	0.540	0.355–0.692
Serum	15	0.538 ± 0.260	0.207–1.229	0.486	0.360–0.733
**Face Cosmetics**					
Cream	46	2.759 ± 2.914	0.082–12.151	1.640	0.214–4.860
Eye cream	11	8.680 ± 9.958	0.202–28.561	3.850	0.383–14.271
Foam	4	0.428 ± 0.392	0.010–0.789	0.456	0.094–0.761
Gel	10	4.384 ± 5.504	0.021–14.260	2.005	0.170–7.964
Hydrolate	3	0.545 ± 0.081	0.482–0.636	0.518	0.482–0.636
Mask	17	1.114 ± 1.601	0.053–6.624	0.525	0.177–1.439
Micellar liquid	9	0.857 ± 0.800	0.003–2.334	0.559	0.294–1.111
Serum	16	5.055 ± 9.235	0.110–37.768	2.168	0.359–5.170
SPF cream	13	1.708 ± 2.351	0.010–8.330	0.970	0.229–2.309
Tonic	5	1.224 ± 1.609	0.036–3.813	0.348	0.145–1.636
Total	268	1.852 ± 3.905	0.003–37.768	0.644	0.315–1.180

Max: maximum; Med.: median; Min: minimum; *n*: number of samples; Q1: quartile 1; Q3: quartile 3; SD: standard deviation.

## Data Availability

Detailed data is available from the authors.
